# Ionic bis-nanoparticle networks

**DOI:** 10.1007/s00706-011-0709-x

**Published:** 2012-01-21

**Authors:** Marie-Alexandra Neouze, Marco Litschauer, Michael Puchberger, Johannes Bernardi

**Affiliations:** 1 Institute of Materials Chemistry, Vienna University of Technology, Vienna, Austria; 2 Transmission Electron Microscopy Centre, USTEM, Vienna University of Technology, Vienna, Austria

**Keywords:** Material science, Heterocylces, Green chemistry, Nanostructure

## Abstract

**Abstract:**

A newly arising challenge in the field of nanoparticle research concerns the control and the understanding of the interparticle interactions and interparticle properties. This should allow the development of materials based on nanoparticle assemblies which represents a great opportunity to exploit nanoparticle collective properties. Although some nanoparticle networks have been reported, few works are addressing the highly exciting problem of forming bis-nanoparticle assemblies in which two different types of nanoparticles are present. In this article we report an original synthesis pathway for the formation of an ionic bis-nanoparticle network, silica/silver, based on the formation of an imidazolium bridging unit. The reaction used for the formation of the bridging imidazolium can be considered as click-like chemistry. The synthesis of the metal/metal oxide hybrid composite material starts from the formation of a metal oxide nanoparticle modified with an imidazole ligand. This composite formation is therefore very general and could be extended to other metal/metal oxide composites.

**Graphical Abstract:**

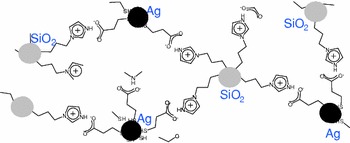
.

## Introduction

The exploration of nanoparticles as building blocks for functional materials led to a better understanding of how to control the size, shape and composition of these nano-objects. The newly arising challenge in the field of nanoparticle research concerns the control and the understanding of the interparticle interactions and interparticle properties. This should allow the development of materials based on nanoparticle assemblies which represents a great approach to exploit nanoparticle collective properties. To address this issue, material chemists started to investigate molecularly linked nanoparticles in order to obtain nanoparticle networks [1–3]. In this context, we recently published the synthesis of ionic nanoparticle networks (INNs), in which metal oxide nanoparticles are covalently bridged by imidazolium units [4]. To form this hybrid organic–inorganic material, the formation of the networks was driven by a nucleophilic substitution occurring between an imidazole functional group and a chloroalkyl group present on the surface of the nanoparticles. To this end, one batch of nanoparticles was modified with imidazole functionalities whereas a second one with chloroalkyl groups. The two batches were then mixed in order for the functionalities to react. This reaction is remarkable owing to three main aspects. First, the nucleophilic reaction can be considered as a click-like chemistry meaning that it is performed in environmentally friendly solvents, such as water or ethanol, at low temperature, at 70 °C, without formation of by-products and with high yield, between 84 and 100%. Second, the proposed procedure can be adapted to various metal oxide nanoparticles, e.g. silica or titania [5, 6]. Third, the synthesis pathway proposed to form INNs allows the possibility of forming bis-nanoparticle networks. The term ‘bis-nanoparticle network’ refers to nanoparticle networks in which two different types of nanoparticles are present. Such bis-nanoparticle assemblies show promise in a wide range of applications, e.g. sensors and catalysis [7–10].

Few works are addressing this highly exciting problem of forming bis-nanoparticle assemblies. Among them, Xu and Ye [11] reported the formation of organic bis-nanoparticle networks obtained by click reaction of azide and alkyne functional groups grafted on to a surface. Mann and co-workers [12] recently proposed a very elegant synthesis route to inorganic bis-nanoparticle networks by using a sodium alginate biopolymer for the formation and stabilization of gold nanoparticles before growing a network of ceria nanoparticles in situ.

In some related works, difunctionalized ligands, carboxylic acid-terminated alkanethiols, were used to first modify a gold surface; the pendant carboxylic acid groups then allowed the possibility to attach to other species in a second step [13, 14]. Nevertheless, in this approach the species alternation can hardly be controlled given that both thiol and carboxylic acid groups can anchor to the metal surface. In addition, in a previous work on silica nanoparticles, we showed that use of difunctionalized ligand, 1,3-di(3-trimethoxysilylpropyl)imidazolium iodide, leads to the formation of poorly extended networks [4, 15].

In this article we report an original synthesis pathway for the formation of an ionic bis-nanoparticle network based on the formation of an imidazolium bridging unit.

## Results and discussion

The starting unit for the synthesis of the bis-INN is the formation of the imidazole-functionalized nanoparticles, as already reported for the synthesis of INN [4]. The silica nanoparticles are modified with imidazole alkoxysilane. The grafting reaction is performed in ethanol.

**Fig. 1:**
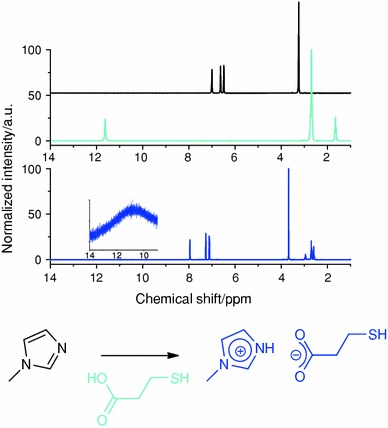
*Top* ^1^H NMR spectra of, from *top* to *bottom*, *N*-methylimidazole, mercaptopropionic acid and thio-imidazolium carboxylate. *Bottom* reaction scheme

The imidazole functional group on the surface of the nanoparticle is then modified to introduce thiol functional groups, which are preferential grafting functionalities for the coordination to metal surfaces [16]. This modification is aided by the basicity of the imidazole groups. On addition of mercaptopropionic acid to the imidazole-functionalized nanoparticle suspension, the acidic protons of the carboxylic acid group will be transferred to the nitrogen of the imidazole unit. This reaction, in the liquid state, so without nanoparticles, can be followed by ^1^H NMR. In the NMR spectrum (Fig. 1, top), the formation of the imidazolium unit is characterized by a 1 ppm shift toward lower field of the aromatic protons. In addition, the carboxylic acid proton of the precursor, around 12 ppm, can no longer be observed, whereas a strongly broadened peak in the same region can be identified, characteristic of a labile, exchangeable proton, as is reported for such imidazolium carboxylate units [17].

The successful use of hydrogen bonds to self-assemble building blocks of various types was recently reviewed [18]. The overall effect of several hydrogen bonds can be very significant and lead to a strong bonding. Concrete examples can be found for the formation of pyrrole-based clusters [19] but also for the formation of supramolecular layers based on the interaction between imidazole and carboxylic acids [17, 20]. This method will then be adapted in order to design bis-nanoparticle networks.

**Fig. 2:**
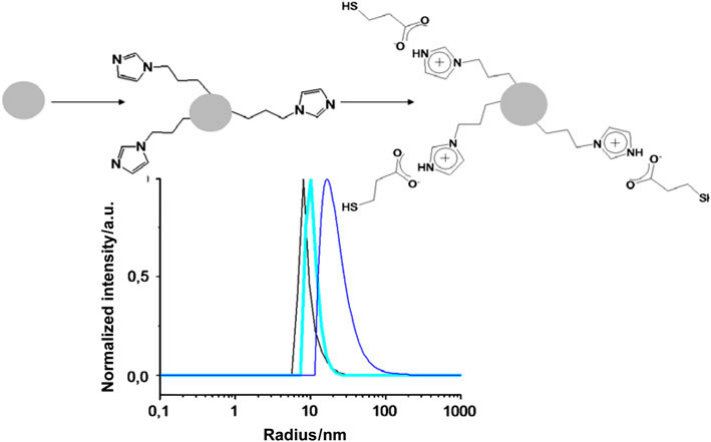
*Top* Scheme of the silica nanoparticle functionalization. *Bottom* DLS of the SiO_2_ nanoparticles from *left* to *right*: non-modified, imidazole functionalized (SiO_2__Im) and thio-imidazolium carboxylate functionalized (SiO_2__ImHOOC_SH)

The reaction described above (Fig. 1, bottom) was then carried out on imidazole-functionalized silica nanoparticles, by adding an equimolar amount of mercaptopropionic acid to the suspension (Fig. 2, top). The nanoparticle suspension was characterized by dynamic light scattering (DLS). The DLS experiments give access to the distribution of hydrodynamic radius, i.e. including the ligand shell and the solvent shell. Thus, at each step of the nanoparticle functionalization, a slight increase of the nanoparticle radius can be observed (Fig. 2, bottom). The radius of the starting “naked” silica nanoparticles is centred on 8 nm, whereas the radius is centred on 11 nm after functionalization with imidazole groups (SiO_2__Im). After addition of the mercaptopropionic acid, the shift toward larger radius is clearer, and the hydrodynamic radius of the thio-imidazolium-modified particles (SiO_2__ImHOOC_SH) is centred on 15 nm.

**Fig. 3:**
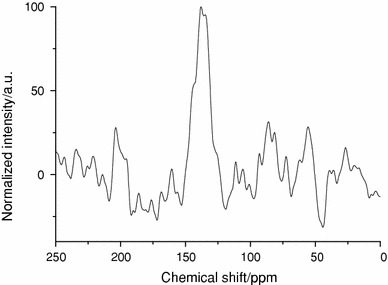
CP MAS ^15^N NMR of SiO_2__ImHOOC_SH

We have shown in previous work on INN that the modification of an imidazole group into an imidazolium can be followed by means of solid-state cross-polarization and magic angle spinning (CP MAS) ^15^N NMR [5]. The nitrogen peak of the imidazolium species (142 ppm) is at lower field than the peak of the imidazole nitrogen atoms (135 ppm). In the case of the thio-imidazolium-modified nanoparticles, the nitrogen peak is centred on 139 ppm (Fig. 3), with a shoulder at slightly lower field. The chemical shift observed for the SiO_2__ImHOOC_SH is nevertheless an indication for the formation of the imidazolium unit. Indeed, the peak is shifted toward lower field, in comparison with imidazole-functionalized nanoparticles. The difference between alkyl imidazolium species previously reported and these thio-imidazolium units comes from the hydrogen bonds between the carboxylate and the imidazolium, which can shift the nitrogen peak. This effect, a strong shift of the nitrogen peak, was reported to be even stronger in solid-state nitrogen NMR [21]. This observation is in good agreement with the large peak at low field, 11 ppm, observed for the NH species in the ^1^H NMR of the liquid thio-imidazolium carboxylate (Fig. 1, inset in the bottom spectrum).

**Fig. 4:**
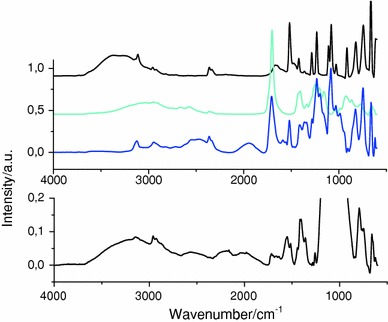
FT-IR spectroscopy of *top* alkyl imidazole, mercaptopropionic acid and alkylimidazolium mercaptopropionate (from *top* to *bottom*), *bottom* SiO_2__ImHOOC_SH

Fourier transform infrared (FT-IR) spectroscopy of the reaction in the liquid state, between an alkyl imidazole and mercaptopropionic acid but without silica nanoparticles, showed the presence of a band at 2,000 cm^−1^ characteristic of the formation of the N–H bond by transfer of the proton from the carboxylic acid group to the imidazole group (Fig. 4, top).

The FT-IR spectrum obtained after similar proton transfer, by reaction of mercaptopropionic acid on the imidazole-modified silica nanoparticles (SiO_2__Im_SH), was measured. Given the very strong absorption band of the Si–O–Si bonds centred at 1,130 cm^−1^, the presented spectrum (Fig. 4, bottom) was zoomed in order to focus on the relatively lower intensity absorption bands. From this spectrum one may deduce the formation of a strong N–H···O hydrogen bond which gave a broad low frequency band around 2,000 cm^−1^ (Fig. 4, bottom). The formation of the band at 2,000 cm^−1^ was reported to be a signature for the N–H···O stretching bands of imidazolium carboxylate and pyridinium carboxylate species [17]. Similar to the results reported in the literature [17], a very broad adsorption between 1,515 and 1,720 cm^−1^ reveals the concomitant presence of carboxylate antisymmetric C–O stretching bands, and at relatively higher wavelength, carbonyl stretching bands. In addition, a broad thiol S–H adsorption band appears at 2,460 cm^−1^ [22, 23].

After obtaining the suspension of thio-imidazolium–carboxylate silica nanoparticles, SiO_2__ImHOOC_SH, we added silver nitrate precursor to the suspension before reduction with sodium borohydride. The hybrid material obtained after reduction will be referred to hereafter as SiO_2__Ag.

**Fig. 5:**
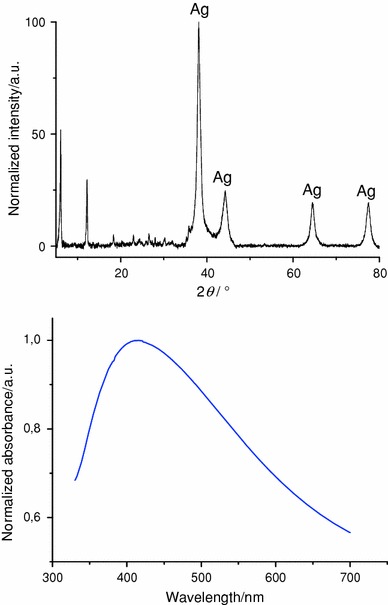
*Top* XRD diffractogram and *bottom* UV–Vis absorption spectrum of the silica/silver bis-nanoparticle network

The X-ray diffractogram obtained for the final hybrid material SiO_2__Ag (Fig. 5, top) confirmed the reduction of the silver cations. The reflections of the final composite at 2*θ* = 38°, 44°, 64.5°, 77.5° and 82° were characteristic of metallic silver. Scherrer’s equation, applied on the silver (111) reflection at 38°, affords an average diameter of silver crystallites in the 14 nm range. In addition, UV–Vis absorption spectroscopy was performed. Indeed, the UV–Vis absorption band of silver nanoparticles can be directly related to the size of the nanoparticles [24]. The absorption centred on 410 nm for the silica/silver bis-nanoparticle network indicated the presence of silver nanoparticles with a size between 10 and 15 nm (Fig. 5, bottom). This characterization confirms that the crystallite size estimated by X-ray powder diffraction (XRD) corresponds also to the size of the nanoparticles contained in the material.

The presence of a sharp so-called pre-peak, at very low diffraction angle, was reported in the X-ray scattering diffractogram of some imidazolium ionic liquids with 6–10 carbon atoms in the alkyl chains and spherical or highly symmetrical anions [25]. The origin of this feature is related to the intermolecular interactions between the imidazolium species and due to the anisotropy of the imidazolium cation. The pre-peak observed refers to the typical distance between charged groups separated by cationic alkyl chains. In the case reported in this article, we assume that the meso-organization of the imidazolium units is forced by the linking on the nanoparticle. Indeed neutral imidazole species are first grafted on to the silica surface. When the imidazole reacts with the carboxylic acid moiety to form charged imidazolium units, even without long mesogen alkyl chains, the charged species are forced, by the grafting, to stay close to one another. This result is in perfect agreement with our previous investigation of short-range order in silica-based nanoparticle networks [26, 27].

**Fig. 6:**
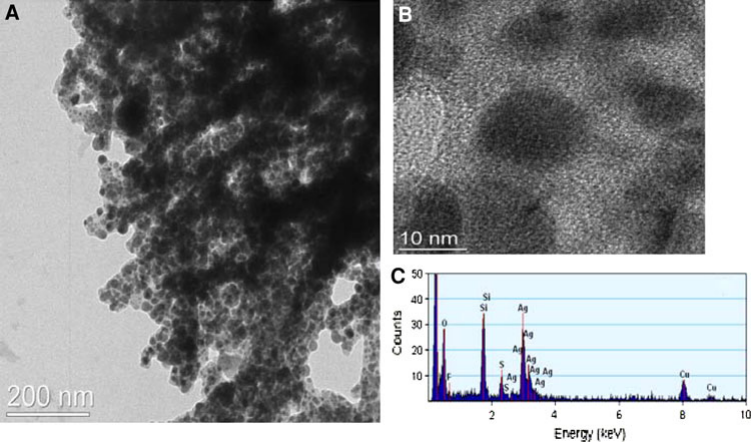
**a**, **b** High resolution TEM micrographs of the silica/silver bis-nanoparticle network and **c** EDX spectrum of the hybrid material

Transmission electron microscopy (TEM) was used to observe the networks of nanoparticles (Fig. 6). However, the alternation of the nanoparticles could not be distinguished given that the material is a three-dimensional structure. Moreover the electron density of the ionic linker and that of the silica nanoparticles are quite similar and make the distinction harder, whereas the crystalline silver nanoparticles could easily be observed (Fig. 6b). Electron dispersive X-ray (EDX) analysis of the region observed in the micrograph in Fig. 6b allowed the various elements contained in the hybrid material to be detected (Fig. 6c), i.e. S, Ag, Si, O, C, the nitrogen atoms are detected as a shoulder at the foot of the oxygen peak and the Cu peak originates from the copper grid used to deposit the hybrid material.

## Conclusion

We have presented an original synthetic procedure for the preparation of metal/metal oxide composite hybrid materials based on silica and silver nanoparticles. The synthesis starts from imidazole-modified nanoparticles which react with mercaptocarboxylic acid to form an imidazolium carboxylate unit with a pendant thiol group. In a further step silver salts are added to the suspension of thiol-modified nanoparticles and reduced. In this procedure, the two-step formation of the bridging unit ensures a selective linking of the nanoparticles. In this way the metal nanoparticles will be neighboured by metal oxide nanoparticles and vice versa.

As for previously reported INNs, the reaction used for the formation of the bridging imidazolium can be considered as click-like chemistry. This reaction occurs in ethanol or water, at low temperature, with no formation of side products.

By characterizing the final bis-nanoparticle hybrid material, it turned out that starting from neutral imidazole-modified nanoparticles allows one to force the imidazolium self-assembly. The mesoscopic arrangement of the imidazolium units could be determined by X-ray diffraction.

By reaction on the pendant imidazole groups, we assume this procedure can be extended to other imidazole-modified metal oxide nanoparticles, like metal nanoparticles of the previously reported imidazole-modified titanium oxide nanoparticles. These imidazole-modified nanoparticles were obtained by grafting of a phosphonic acid imidazole ligand on to the surface of the titanium oxide nanoparticles.

## Experimental

### Chemicals

Chemicals were used without further purification, unless otherwise stated. Imidazole, 3-mercaptopropionic acid, dry methanol, *N*-methylimidazole, silver nitrate, sodium borohydride, sodium hydride and sodium iodide were obtained from Sigma-Aldrich. Tetraethyl orthosilicate (TEOS) was obtained from Fluka. Aqueous ammonia solution (32%), ethanol and tetrahydrofuran (THF) were obtained from Merck. 3-Chloropropyltrimethoxysilane was obtained from ABCR, acetone from Fisher Scientific and dichloromethane from Riedel-de Haën.

### Characterizations

Solid-state NMR spectra were recorded on a Bruker AVANCE 300 (^1^H at 299.85 MHz, ^13^C at 75.40 MHz, ^11^B at 96.21 MHz, ^31^P at 121.38 MHz and ^15^N at 30.38 MHz) equipped with a 4 mm broadband MAS probe head. ^13^C and ^15^N spectra were recorded with ramped CP MAS experiments. The sample holders were spun at 6 kHz for the ^31^P, ^15^N and ^13^C experiments and at 10 kHz for the ^11^B measurements. Liquid-state NMR spectra were recorded either on a Bruker AVANCE DPX 300 (^1^H at 300.13 MHz, ^13^C at 75.47 MHz), equipped with a 5 mm broadband inverse probe head or on a Bruker AVANCE 250 (^1^H at 250.13 MHz, ^13^C at 62.90 MHz, ^31^P at 101.25 MHz), equipped with a 5 mm QNP probe head. ^1^H/^15^N heteronuclear multiple-bond correlation (HMBC) spectra were measured with long-range coupling constants of 5 Hz or 10 Hz.

The samples for TEM measurements were prepared by dispersing the particles in ethanol prior to deposition on a carbon-coated TEM Cu grid. TEM measurements were performed on an analytical TECNAI F20 field emission TEM system connected to an EDX detector (USTEM, Vienna University of Technology). XRD measurements were performed on a Philips X’Pert diffractometer using the Cu–Kα radiation (*λ* = 1.542 Å).

For the DLS measurements, the solid was dissolved in ethanol. DLS experiments were carried out without previous sonication of the samples. The run time of the measurements was 10 s. Every size distribution curve was obtained by averaging ten measurements. The apparatus was an ALV/CGS-3 compact goniometer system, equipped with an ALV/LSE-5003 light scattering electronics and multiple τ digital correlator, and a 632.8 nm JDSU laser 1145P.

To record the FT-IR spectra, the products were measured using an ATR unit (ZnSe crystal for liquids, diamond for powder samples). The spectrometer was a Bruker Tensor-27-DTGS equipped with an Interferometer RockSolidTM and a DigiTectTM detector system, high sensitivity DLATGS, using the OPUSTM software.

The UV–Vis spectroscopy measurements were carried out on a PerkinElmer Lambda 35 with a scan speed of 480 nm/min and a slit width of 1 nm.

### Syntheses

The syntheses of the silica nanoparticles and the ligand, *N*-imidazolylpropyl trimethoxysilane (Si–Im), were reported elsewhere [4, 27].

#### *N*-*Methylimidazolium mercaptopropionate*

In a small beaker 1 g of *N*-methylimidazole (12.18 mmol) was mixed with 1.290 g 3-mercaptopropionic acid (12.18 mmol). During the addition an exothermic reaction occurred. The clear solution was stirred overnight.

#### *Modification of SiO*_*2*_* nanoparticles withN-imidazolylpropyltrimethoxysilane* (SiO_2__Im)

60 cm^3^ of the previously prepared silica nanoparticle suspension, obtained by reacting 15 mmol of tetraethoxysilane, was transferred into a 250-cm^3^ round-bottom flask and degassed in a vacuum for several minutes to remove excess ammonia. Afterwards 1.152 g of *N*-imidazolylpropyl trimethoxysilane (5 mmol) were added dropwise. The solution was stirred under argon at room temperature overnight.

#### *Thiol functionalization* (SiO_2__Im_SH)

To the above prepared solution of imidazole-functionalized silica nanoparticles (imidazole/thiol = 1:1) 0.531 g of 3-mercaptopropionic acid (5 mmol) was added. The solution was stirred overnight.

#### *Formation of the bis-nanoparticle network: growing of the silver nanoparticles* (SiO_2__Im_Ag)

To 20 cm^3^ of the above prepared solution of thio-imidazole-functionalized silica nanoparticles 1.032 g silver nitrate (6.1 mmol) dissolved in 50 cm^3^ water was added (thiol-imidazole/Ag^+^ = 1:4). A white suspension formed immediately. Afterwards 0.252 g sodium borohydride (6.1 mmol) dissolved in 25 cm^3^ water was added within 30 s. A violent reaction occurred and a precipitate formed immediately. The suspension was allowed to stir overnight at ambient temperature. Afterwards the product was obtained by centrifugation and washing twice with ethanol, water and acetone successively. Finally the powder was dried under reduced pressure (2 mbar) at 80 °C.
